# Relationships between infant mortality, birth spacing and fertility in Matlab, Bangladesh

**DOI:** 10.1371/journal.pone.0195940

**Published:** 2018-04-27

**Authors:** Arthur van Soest, Unnati Rani Saha

**Affiliations:** Department of Econometrics and Operations Research, Tilburg School of Economics and Management, Tilburg University, LE Tilburg, The Netherlands; Center for Healthy Start Initiative, NIGERIA

## Abstract

Although research on the fertility response to childhood mortality is widespread in demographic literature, very few studies focused on the two-way causal relationships between infant mortality and fertility. Understanding the nature of such relationships is important in order to design effective policies to reduce child mortality and improve family planning. In this study, we use dynamic panel data techniques to analyse the causal effects of infant mortality on birth intervals and fertility, as well as the causal effects of birth intervals on mortality in rural Bangladesh, accounting for unobserved heterogeneity and reverse causality. Simulations based upon the estimated model show whether (and to what extent) mortality and fertility can be reduced by breaking the causal links between short birth intervals and infant mortality. We find a replacement effect of infant mortality on total fertility of about 0.54 children for each infant death in the comparison area with standard health services. Eliminating the replacement effect would lengthen birth intervals and reduce the total number of births, resulting in a fall in mortality by 2.45 children per 1000 live births. These effects are much smaller in the treatment area with extensive health services and information on family planning, where infant mortality is smaller, birth intervals are longer, and total fertility is lower. In both areas, we find evidence of boy preference in family planning.

## 1. Introduction

According to the demographic transition theory, there is a strong correlation between childhood mortality and fertility, with an important role for birth spacing in shaping this relationship. Understanding the empirical nature of such relationships is important to design effective policies for reducing child mortality and fertility. The importance of this is illustrated by, for example, United Nations Sustainable Development Goal 3.2: “By 2030, end preventable deaths of newborns and children under 5 years of age, with all countries aiming to reduce neonatal mortality to at least as low as 12 per 1,000 live births and under-5 mortality to at least as low as 25 per 1,000 live births” [1]. Empirical evidence has shown that a decline in childhood mortality is often a prerequisite for fertility decline [2–4], while other studies have emphasized causal links in the reverse direction: high fertility and the close birth-spacing associated with it cause an increase in child mortality [5–6]. Yet another set of studies emphasized that the analysis of the direction of causality is hampered by the existence of several interrelations between child mortality, birth intervals, and fertility at the same time [7–8].

The observed associations between child mortality, birth spacing, and fertility may not only be due to causal mechanisms but can also be explained by common unobserved factors (omitted variables or confounding factors) that drive the various processes. To evaluate policies aimed at optimal birth spacing, reducing mortality, and reducing fertility, identifying the importance of the various causal mechanisms and alternative explanations is crucial. If associations reflect spurious correlation or reverse causation instead of the presumed causal effects, then the policy implications can be dramatically altered [9–10].

One of the most important policy parameters in this context is the replacement effect: how does mortality affect total fertility? In the seminal work of Olsen, the issue was raised that straightforward estimates of this effect can be biased because mortality will also be driven by fertility decisions, for example through birth intervals [11]. Olsen developed a new and simple method to study replacement effects, also applied in [12], for example. It was also pointed out, however, that substantial within-parity variation in mortality rates may hamper the application of Olsen’s method [12–13].

An alternative to the Olsen approach is the multivariate panel data framework developed by Bhalotra and van Soest that models all mortality-fertility causal relationships within each family [14]. This type of model addresses the concerns raised in the recent study on demographic analysis conducted in Matlab, Bangladesh [15], who emphasize the importance of jointly modelling child deaths, interval lengths and fertility behaviour, while allowing for correlated death risks among different births to the same mother.

In this study, we use a panel data model similar to the one introduced in [14] to analyse infant mortality, birth intervals, and fertility in Matlab, Bangladesh. This model captures the relevant causal mechanisms and accounts for confounding factors through potentially correlated unobserved heterogeneity terms in the death risks and fertility and birth interval decisions. It exploits the timing of the sequence of all births and deaths to a mother for identification. It has equations for mortality (of each child born in a given family), for whether there will be a next birth, and for the birth interval. Mortality depends on, among other things, the length of the preceding birth interval (for birth orders higher than one), age of the mother at childbirth, gender of the child, socio-economic status of the family, religion, and an unobserved mother specific effect. Whether or not there will be a next birth and the interval after a given birth until the next birth in turn depend on gender and survival status of previously born children, age of the mother at childbirth, socio-economic status, religion, and unobserved mother-specific effects. The three mother-specific unobserved effects are allowed to be correlated to capture the possibility of common unobserved factors driving the various processes. The model is estimated with maximum likelihood, accounting for all the correlations and for censoring in the birth spacing equation (due to the limited observation window). The estimates therefore remain consistent in spite of the endogeneity of some of the explanatory variables.

The main novelty compared to [14] is that our application concerns infant mortality in rural Bangladesh. While Bhalotra and van Soest used retrospective data to analyse neonatal mortality in India, we use prospective data from the Demographic and Health Surveillance System, Matlab, following mothers residing in the study area over time. This has the advantage that several covariates, such as indicators of socio-economic status and environmental factors such as availability of drinking water are observed at the relevant points in time when children are born (rather than at survey time in retrospective data). Moreover, it avoids recall error in, for example, the dates when children were born.

Another specific feature of our data is that the study area is randomly split into villages with standard government provided health services (the “comparison area”) and villages with additional extensive health and family planning programmes, such as more health clinics and regular visits of health officers (the “icddr,b area” or “treatment area”) [16–17]. Due to the many differences between the areas, differences cannot be ascribed to specific interventions, but they do provide insight in how the complete set of extensive health and family planning services affects the replacement effects and the other links between mortality, birth spacing, and fertility.

### Background and existing studies

There is an extensive literature of reduced form studies on the associations between mortality, birth intervals, and fertility, and we do not have the ambition to review this literature. Studies typically found a strong negative association between child mortality and subsequent birth intervals and, accordingly, a positive relationship between child mortality and the number of subsequent births, especially in developing countries. For Bangladesh, for example, it was found that infant death is associated with a reduction of the median of the subsequent birth interval from 37.2 to 24.1 months [2]. Following the approach of [11,12], concludes that in China, for every infant death, 0.6 extra children were born- and this rate is three times higher than the rates in Colombia and Malaysia [11, 18].

According to the classical demographic transition theory, child mortality affects fertility mainly in two ways: physiological/biological changes and volitional /replacement effects. The physiological effect can be explained by the fact that breastfeeding is interrupted with a child death, shortening the postpartum infecundable period [19]. As a result, the mother is able to conceive the next child sooner, possibly leading to a shorter birth interval and higher fertility. Volitional or replacement effects occur when couples seeking a desired family size replace a lost child. It is difficult to distinguish the physiological and volitional replacement effects empirically [13]. The current study aims to estimate the total mortality effects (either physiological or volitional) on fertility via reducing birth intervals, and vice-versa.

An alternative way in which mortality may affect fertility is hoarding [9, 20]. Hoarding refers to the fertility response to *expected* mortality of the offspring, while replacement is the response to an *actual* child death. Hoarding is not expected to be very important in the current setting, since women can usually respond to the realized infant death by having another birth.

Many studies also found an association between a short birth interval and infant death of the *next* child, particularly when the preceding sibling survived [21–23]. An explanation for this is that the mother has not recuperated physiologically from the previous birth [24–25]. Hence vulnerable families can be caught in a death trap that leads to clustering of child deaths within families: the death of a child reduces the interval to the next birth and thus increases in the risk of death of the subsequent sibling in the family [26]. An alternative explanation suggested is that a child death leaves the mother depressed [27]. This may affect the mother’s behaviour, compromising the health of her subsequent child in the womb and in early infancy. Sibling competition may also explain why short birth intervals and high fertility increase death risk: sources of food and care per head diminish as the number of dependent members of a family increases [5]. This would induce a negative effect of child death on the mortality risk of the next child, since the next child competes with fewer siblings [23]. A negative effect may also be due to learning: If the older sibling died due to, for example, diarrhoea or acute respiratory infections (ARI)–two leading causes of child death explaining almost half of all deaths in Bangladesh [28]—the mother may want to learn how to prevent such a death for future births.

Couples continue childbearing until they reach their desired family size and composition. It was found that in Bangladesh, the median birth interval after the death of a child is shorter when the deceased child was a boy or when the family has at most one boy [29]. In Ghana, the probability of having a next birth within a given time period is one third higher if a male child died than if a female child died [30].

Observed clustering in infant or child mortality of successive children may also be due to unobserved confounding factors instead of causal mechanisms. Older studies of birth spacing and childhood mortality usually do not control for both. More recent studies for India and Kenya reveal that the causal effect on child mortality of mortality of the previous sibling is overestimated when unobserved heterogeneity is not accounted for [26, 31]. The model used by Bhalotra and van Soest that is the starting point in this study extends their model by incorporating fertility and birth intervals, so that the mechanisms discussed above can all be analysed within one coherent modelling framework.

## 2. Data

Since 1966 icddr,b maintained a Health and Demographic Surveillance System (HDSS) in Matlab, aiming to support the Bangladesh Health and Family Planning programme. In Matlab, an area located about 60 km southeast of Dhaka, all births, deaths, causes of deaths, pregnancy histories, migrations in and out of the area, marriages, divorces, and several indicators of socioeconomic status are recorded for the complete population of about 220,000 people. The HDSS data on the timing of pregnancy outcomes and deaths are considered to be of very high quality because they are collected during regular visits (every two weeks until the late 1990s and every month since then) by well-trained female community health workers [32, 17]. Villages in the Matlab area were split in two groups: a “comparison area” with standard government provided health and family planning services, and a “treatment area”, usually referred to as “icddr,b area” with many health and family planning interventions introduced over the study period [33–34]. We combined the health and demographic surveillance system data from 70 villages in the icddr,b area and 79 villages in the comparison area obtained from 1 July, 1982 until 31 December, 2005 (the study period). Data from before 1 July 1982 have not been made available for research.

The complete data set has records on about 63,000 mothers, with more than 165,000 childbirths, including multiple births and stillbirths. We eliminated mothers with multiple births (3401 births in both areas together) as children of a multiple birth face much higher odds of dying and require a separate analysis (as documented in the demographic literature). We also eliminate mothers with incomplete birth history information. For example, if a mother has had four live births, she should appear four times, with four recorded birth dates. In all other cases e.g., if a child was born outside Matlab or before the study period, we do not have the required information on this child, and had to delete the mother from the sample. Similarly, we deleted mothers who migrated out of Matlab during the period under study. The sample therefore only contains mothers who continuously lived in the Matlab area since the birth of their first child until the end of the study period.

Moreover, we have excluded the children born in three villages that shifted from the icddr,b area to the comparison area in 2000. These sample selection steps lead to a sample in the comparison area of 32,366 children out of the original raw sample of 74,214 and 11,856 mothers out of the original 30,264. In the icddr,b area the working sample has 31,968 children (out of 67,696) and 13,232 mothers (out of 32,391). We acknowledge that these sample selection criteria imply that our results are not necessarily representative for the complete population of the Matlab area. Studying the relation between child mortality and, for example, migration into or out of Matlab, is a topic for future research.

Furthermore, we discarded stillbirths (11,990 stillbirths are recorded in the comparison area and 8,646 in the icddr,b area). One reason for this is that gender, an important covariate in our analysis, is missing for stillbirths. Accordingly, we define birth intervals as intervals between reported dates of live births, ignoring stillbirths in between live births. This may imply that some long birth intervals are due to stillbirths. Distinguishing stillbirths from other mechanisms leading to long birth intervals is another topic left for future research. Since stillbirths then have to be modelled, it will be an additional layer of complexity in an already rather complex model. This is also that our study fails to address local community (village) specific effects. On the other hand, we do not have any reason to expect that by excluding stillbirths this induces a systematic selection bias in a specific direction lengthen birth intervals. For example, stillbirths are more common among mothers in the comparison area compared to the icddr,b area; however, the length of birth intervals is longer in the icddr,b area. Future study may address more rigorous analysis by handling the potential bias.

Table 1 presents sample means or percentages of outcome 1 (for dummy variables) by area. In the comparison area, 6.82 percent of all children died during infancy -8.90 percent among first born and 5.62 percent among higher order births. Of all families, 15.66 per cent experienced at least one infant death and 1.08 per cent lost all their children. About 20.65% of all birth intervals are shorter than or equal to 24 months. In the icddr,b area, birth intervals tend to be longer and infant mortality is less common: 5.09 percent of all live births; 10.66 percent of all families experienced at least one infant death. The average number of children born per mother is 2.73 in the comparison area and 2.42 in the icddr,b area; 29 percent of families had more than three children in the comparison area, compared to 19 percent in the icddr,b area (not reported in the table). Due to the interventions in the icddr,b area, mothers in that area more often have access to a more hygienic source of drinking water (with an underground tube well or a filter) and live closer to a health facility.

**Table 1:** Descriptive statistics by area, Matlab, 1982–2005.

Variables	icddr,b area	Comparison area
Infant deaths (all live-births) (%)	5.09	6.82
Infant deaths excluding first-borns (%)	3.95	5.62
Infant deaths among first borns (%)	6.70	8.90
Families with no infant deaths (%)	89.34	84.34
Families in which all births die in infancy (%)	0.79	1.08
Preceding birth interval in months (%)		
< = 24 months	12.93	20.65
25–36 months	19.92	32.73
> = 37 months	67.14	46.63
Age of mother at first birth*	21.16 (3.23)	21.08 (3.21)
Age of mother at birth*	24.70 (5.03)	24.58 (4.85)
**Mother’s education level (%)**		
No education	48.48	50.50
Some primary education	24.86	25.51
At least some secondary education	26.66	23.99
Mother Muslim (%)	82.71	89.85
Child male (%)	50.97	51.12
Birth order (%)		
1	41.39	36.63
2	28.93	26.74
3	17.62	18.26
4+	12.06	18.36
**Father’s education level (%)**		
No education	55.67	56.28
Some primary education	22.65	24.15
At least some secondary education	21.68	19.57
Father day labourer (%)	19.61	20.96
Hygienic drinking water (tube well/piped) water) (%)	87.76	76.91
Distance to health facility (km) *	1.87 (0.98)	7.07(4.04)
Number of mothers in sample	13,232	11,856
Number of children in sample	31,968	32,366

*: continuous variable; other variables are dummy variables. Means of continuous variables, with standard deviation in parentheses; percentage with outcome 1 for dummy variables. No education = 0 years of schooling, some primary education = 1–5 years of schooling, and at least some secondary education = 6 or more years schooling. *Source*: Matlab DSS data.

**Fig 1:**
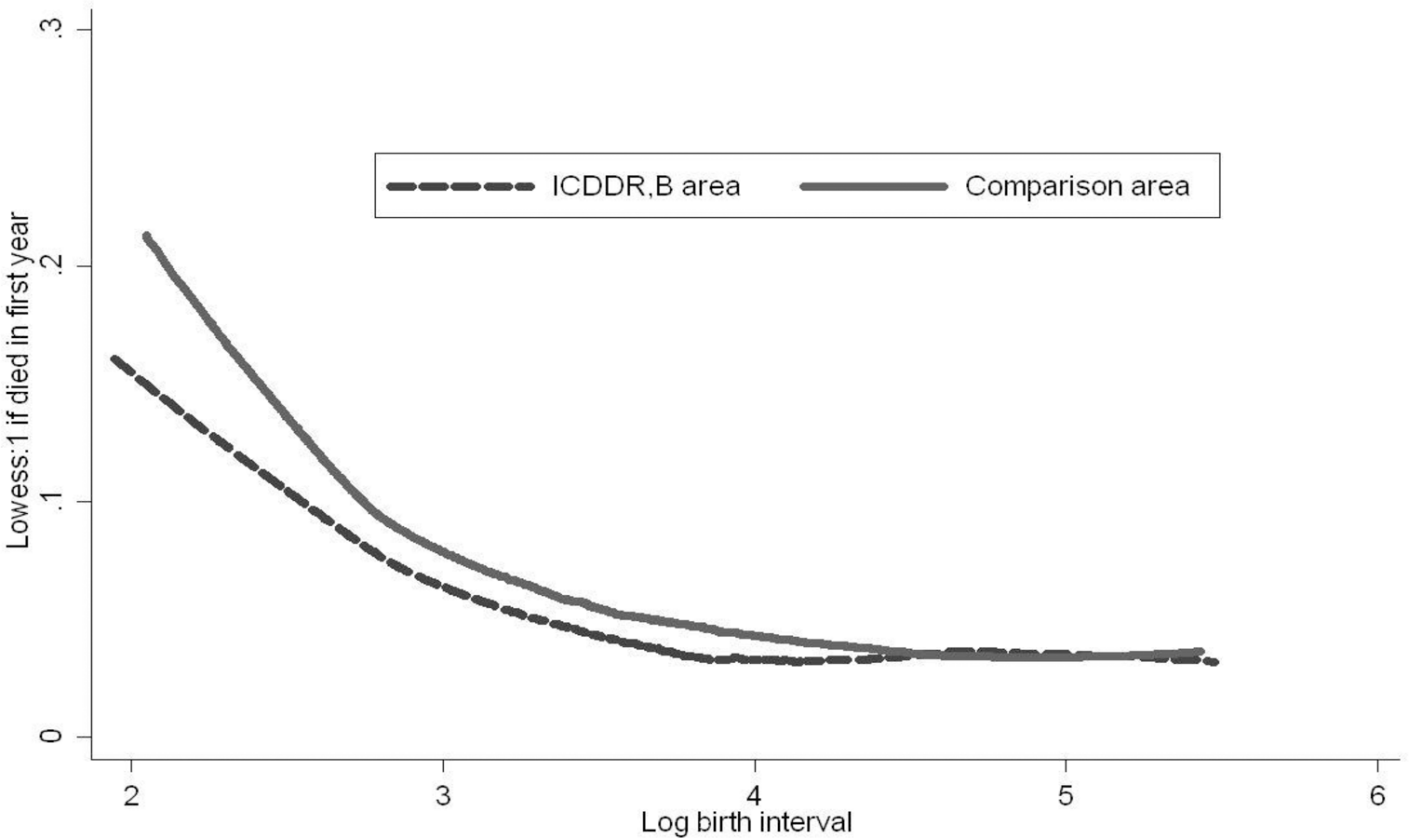
Infant mortality and preceding birth interval. Note: lowess uses the Stata command for local weighted (nonparametric) regression, with default settings.

The non-parametric regressions of infant mortality on the preceding birth interval in Fig 1 show a sharp decline in infant mortality rates when birth intervals increase in both areas. The probability of infant death falls with birth interval length until an interval length about 4.5 years (exp(4) = 54 months). Particularly in the icddr,b area, the survival chances stabilize or even increase somewhat when birth intervals increase beyond 4.5 years.

**Fig 2:**
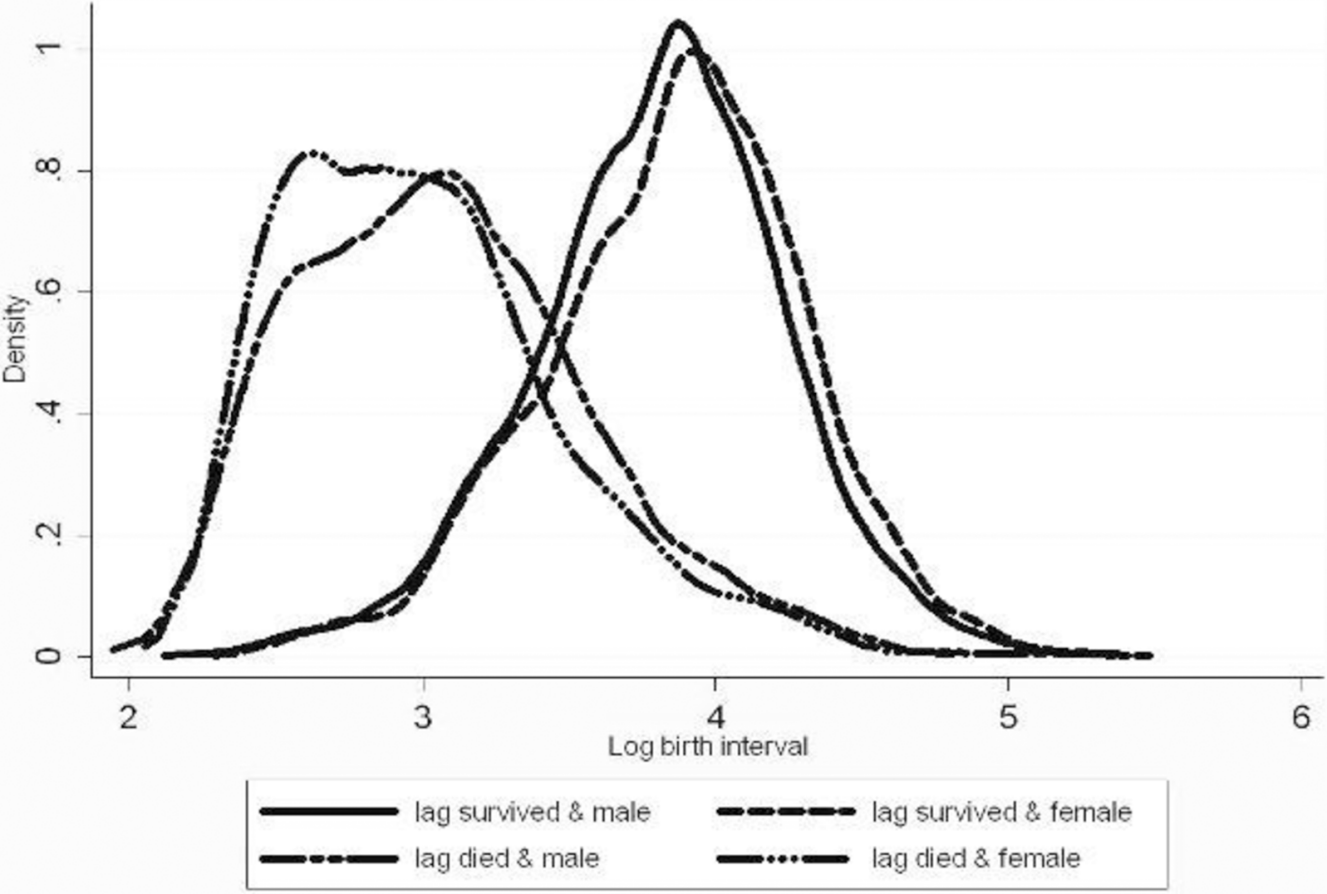
Birth intervals by survival status and gender of previous child, icddr,b area.

**Fig 3:**
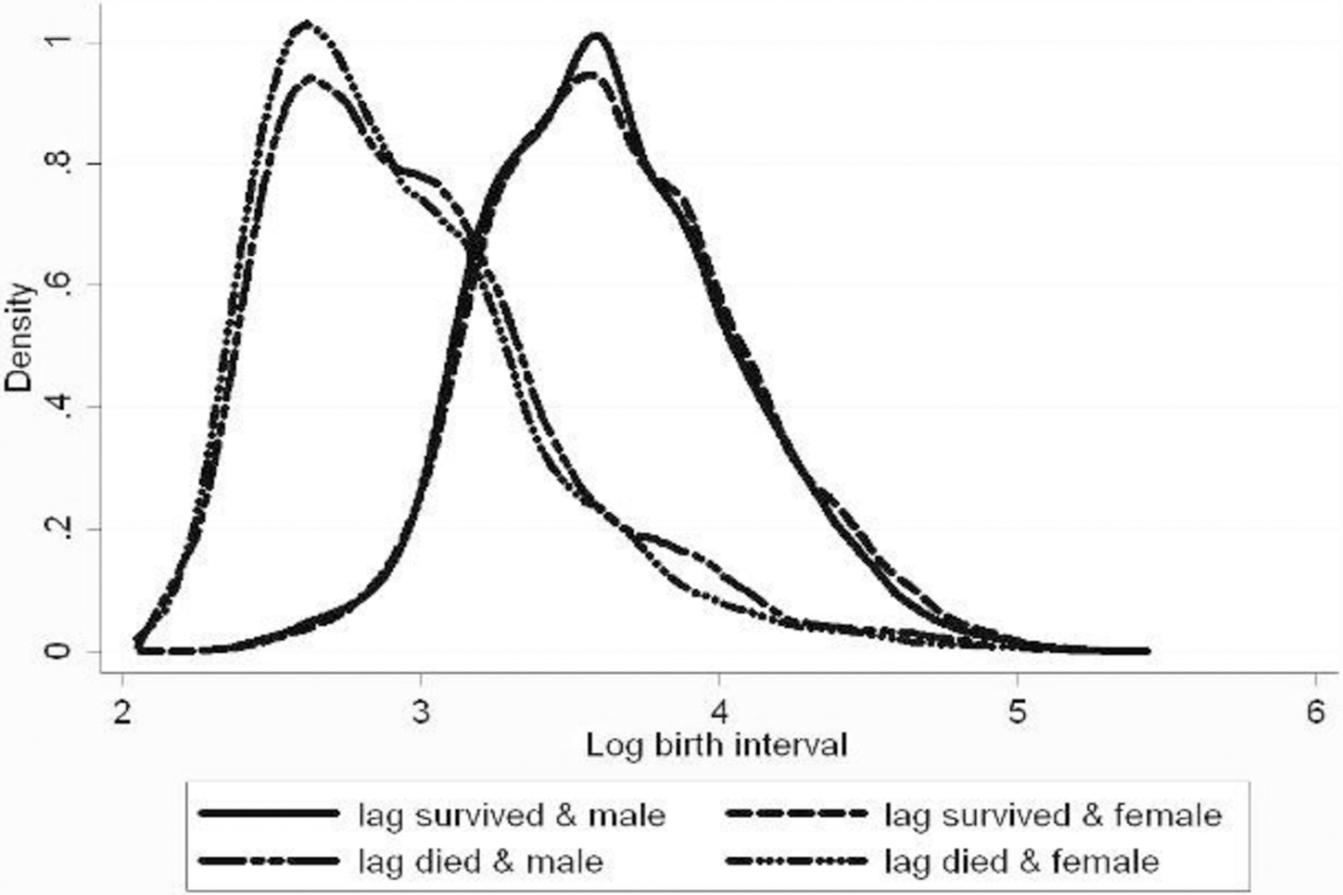
Birth intervals by survival status and gender of previous child, comparison area.

Figs 2 and 3 show the distributions of the log birth interval by survival status of the previous child and by gender. In both areas, there is a large difference between the distributions after infant death and infant survival. In the comparison area, the medians are 17 and 37 months (and the averages are 22 and 42 months). Gender differences are insignificant: the p-values for the mean and median gender differences are 0.370 and 0.706 for the comparison area and 0.328 and 1.000 for the icddr,b area (where the medians coincide).

## 3. Model specification

The model is similar to the one used in [14]. A difference is that we do not consider local community effects. The sensitivity analysis in [14] suggests that this has no effect on the point estimates (though it may mean that our standard errors are somewhat underestimated). Admittedly, the finding in [14] could be specific to their sample of Indian mothers, but given the additional complexity that adding community effects would involve, we did not pursue this extension here.

Conceptually, the model is based upon the notion that a family’s mortality outcomes, birth intervals, and fertility are determined partly by decisions of the family and partly by external factors that can vary over time (e.g., random health shocks) or are persistent over the complete observation period (e.g., genetic factors). We do not explicitly distinguish between external factors and factors controlled by the family and do not aim to work with a fully structural model in which the family solves a dynamic optimization problem that determines its family planning and health behaviour. Instead, we formulate separate equations for the various outcomes, accounting for observed and unobserved time persistent and time varying variables that may affect each of the outcomes, and allowing each of the outcomes to depend upon previous outcomes in the same family. For example, the interval to the next birth may depend upon gender and mortality outcomes of the previous births, the mortality outcome may depend upon the preceding birth interval, etc. This exploits the timing of events (there is no causal effect of future outcomes on current outcomes) to identify of the econometric model. Confounding factors, such us unobserved family attitudes towards modern health behaviour and family planning, are allowed for as long as they are persistent over the mother’s reproductive period, and are captured in unobserved heterogeneity terms that can be correlated across equations.

The benchmark model taken from [14] has equations for the infant mortality outcome of each birth, for the decision to have another child, and for the birth interval. In principle, an equation for the decision to have another child is not necessary, since no next birth corresponds to a birth interval that extends beyond the reproductive age limit. We therefore also consider a version of the model without this equation. Since the complete (benchmark) specification outperforms this simpler specification, in line with the notion that families use other ways of family planning than methods that increase birth intervals, we focus on the results of the complete model.

To be more precise, the endogenous variables in the model are the following, with *i* denoting a mother and *t* = *1*,..,*T*_*i*_ denoting her consecutive live births:

*M*_*it*_: Infant mortality dummy: 1 if child *t* dies; 0 if it survives the first twelve months after birth.

*F*_*it*_: Having a next birth (1) or not (0) after birth *t*.

*B*_*it*_: Log birth interval preceding birth of child *t* (*t>1* only)

**Fig 4:**
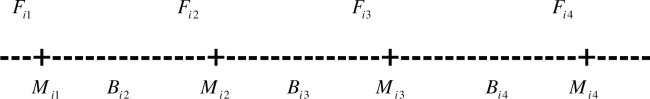
The sequence of events.

The sequence of events is illustrated using the time line in Fig 4:

The timing of the first birth is taken as given. Our data do not provide date of marriage so we do not observe the interval from marriage to first birth. The first event we explain is infant survival of the first born child *M*_*i*1_. The second is the event whether there will be a next birth (*F*_*it*_ = 1) or not (*F*_*it*_ = 0). This is not always observed—if a second birth is observed in the data we know that *F*_*it*_ = 1. If not, this can be because *F*_*it*_ = 0 or because the next birth interval is too long in the sense that it exceeds the observation window or the woman’s reproductive span (set to 45 years of age).

If *F*_*it*_ = 1 and the birth interval is short enough to have the next birth within the observation window, we observe the second birth and the birth interval *B*_*it*_. The second born child can die during infancy or survive; depending on this outcome, there will be a next birth or not (*F*_*i3*_), etc.—the sequence of events continues until the mother has no more children (*F*_*iT*_ = 1), at the end of her fertile period (age 45), or at the end of the observation window (December 2005).

We use probit equations for the binary outcomes (infant mortality of each child; fertility decision after each birth) and a regression equation for the continuous outcomes (log birth intervals). Below we discuss the equations for the various outcomes in more detail.

### Infant mortality

For higher birth orders, a dynamic probit equation with (random) mother specific effects is used. The explanatory variables include the preceding birth interval and variables related to the mother’s age at birth, which is a function of previous birth intervals: For child *t* (*t* = *2*,*…*,*T*_*i*_) of mother *i*, the equation is

$$M_{it}^{*}=X_{it}\beta_{m}+Z_{it}\gamma_{m}+\alpha_{mi}+u_{mit}$$ (1)

$$M_{it}=1\;if\;M_{it}^{*}>0\;and\;M_{it}=0\;if\;M_{it}^{*}\leq 0$$

Here *X*_*it*_ contains (functions of) the strictly exogenous variables, such as gender of the child, socio-economic status indicators of the household (mother’s and father’s education, etc.) and characteristics of the village where the household resides. *Z*_*it*_ is the vector of explanatory variables that are functions of previous outcomes (and are therefore not strictly exogenous), including the preceding log birth interval *B*_*it*_, (functions of) age of the mother at birth *t* and, following the literature on scarring and in particular important work by Arulampalam and Bhalotra, survival status of the previous child *M*_*it-1*_ [26]. The mother specific unobserved heterogeneity term *α*_*mt*_ captures unobservable time invariant characteristics influencing the infant mortality risk of all children in the family. The error term *u*_*mit*_ captures idiosyncratic health shocks specific to child *t*. We assume that the *u*_*mit*_ follow a standard normal distribution, independent of each other and of all covariates, and that *α*_*mt*_ is normally distributed with mean 0 and variance *σ*^*2*^*(α*_*m*_*)* independent of all *u*_*mit*_ and *X*_*it*_ (but not of *Z*_*it*_). Note that Eq (1) does not include the next birth interval. The reason is that we only consider infant mortality, and since 98% of all birth intervals are larger than one year, birth intervals typically extend beyond the infancy period. If a birth is followed by a short birth interval, this may reduce the survival chances of the child that is born later on due to sibling competition, but rarely already during the period of infancy. For mortality of the first child, a separate equation is needed, since there is no preceding birth interval or preceding mortality outcome. Age at first birth is assumed to be exogenous and included in *X*_*it*_. The equation for the first child’s infant mortality is then given by:

$$M_{i1}^{*}=X_{i1}\beta^{1}+\theta \alpha_{mi}+u_{mi1}$$ (2)

$$M_{i1}=1\;if\;M_{i1}^{*}>0\;and\;M_{i1}=0\;if\;M_{i1}^{*}\leq 0$$

Here *β*^*1*^ and *θ* are (auxiliary) parameters to be estimated and the error term *u*_*mi1*_ is assumed to satisfy the same assumptions as the other *u*_*mit*_.

### Birth-spacing

For a mother who has given births to *T(i)* children, we observe the exact log durations in between two consecutive births *b*_*i2*_, …., *b*_*iT(i)*_, preceding births 2, …, *T(i)*. We model these intervals using the following equation:

$$b_{it}=X_{it}\beta_{b}+{Z(b)}_{it}\gamma_{b}+\alpha_{bi}+u_{bit}$$ (3)

Here *X*_*it*_ denotes the vector of strictly explanatory variables, as before. Another determinant of birth spacing would be the use of contraceptives. We do not include this since it is not observed in the comparison area and may be correlated with unobservables in the model. *Z(b)*_*it*_ includes survival status of the preceding sibling and family composition variables (functions of the numbers of surviving girls and boys). We assume gender of each birth is exogenous and thus do not incorporate the possibility of selective abortion. Descriptive statistics confirm that this is not an issue in these data, revealing no relation at all between gender of a newborn child and gender composition of the older children in the family.

The unobserved heterogeneity term *α*_*bi*_ captures unobserved time invariant characteristics of the mother (or her household or village) influencing the birth interval. The error term *u*_*bit*_ captures idiosyncratic errors. We assume that the *u*_*bit*_ follow a normal distribution, independent of each other and of all covariates, and that *α*_*bi*_ is normally distributed independent of all *u*_*bit*_ and *X*_*it*_ (but not of *Z(b)*_*it*_).

### Having a next child or not and right censoring

There is right-censoring in the data since some mothers will not have completed their reproductive span at the time of the survey. After the end of the observation window (ultimo 2005), some mothers will still have another birth, and others will not. In principle, this could be captured with a birth interval after the last observed birth that lasts longer than until end 2005. Following [14], however, we add a separate equation reflecting the possible decision to stop having children after each birth. This improves the fit since it can explain why some mothers who are still of reproductive age (assumed to be 45 years of age—an age beyond which very few births are observed in our data) have no more births long before the end of the observation window. Without the additional equation, this would have to be explained by a very long birth interval; we will compare the fit of the complete model with that of the simpler model without this equation in the next section.

The equation determining whether, after birth *t*, the woman gives at least one other birth (*F*_*it*_ *= 1)* or not (*F*_*it*_ *= 0)* is specified as follows:

$$F_{it}^{*}=X_{it}\beta_{f}+{Z(f)}_{it}\gamma_{f}+\alpha_{fi}+u_{fit}$$ (4)

$$F_{it}=1\;if\;F_{it}^{*}>0\;and\;F_{it}=0\;if\;F_{it}^{*}\leq 0$$

As before, *X*_*it*_ denotes the vector of strictly exogenous explanatory variables. The vector *Z(f)*_*it*_ includes survival status of the preceding sibling and family composition variables (based upon the number of surviving girls and boys). The mother specific unobserved heterogeneity term +*α*_*fi*_ captures unobservable time invariant characteristics influencing the probability to have a next birth and the term *u*_*fit*_ captures idiosyncratic errors. We assume that the errors *u*_*fit*_ are standard normally distributed, independent of each other and the *X*_*it*_. The mother specific unobserved heterogeneity terms *α*_*fi*_ are normally distributed with mean 0 and variance *σ*^*2*^*(α*_*f*_*)*, independent of all *u*_*fit*_ and *X*_*it*_.

The outcome *F*_*it*_ is observed only partially. If birth *t* is not the last birth (*t<T*_*i*_) then we know that the mother will have at least on more birth, so that *F*_*it*_ = 1. But if *t = T*_*i*_, she may have decided to stop having children (*F*_*it*_ = 0), but it may also be the case that the next birth interval extends beyond reproductive age or the end of the observation window (*F*_*it*_ = 1 and right censoring). The data on actual births and birth intervals are not informative about this distinction so that *F*_*it*_ is observed only partially.

Confounding unobserved factors are controlled for by allowing arbitrary correlations amongst *α*_*fi*,_ *α*_*mi*_, and *α*_*bi*_. We will assume they are drawn from a three-dimensional normal distribution with zero mean and an arbitrary covariance matrix, independent of the *X*_*it*_ and the error terms *u*_*fit*,_ *u*_*mit*_, and *u*_*bit*_.

## 4. Model selection and estimation results

The equations of this model (Eqs 1–4) are estimated jointly using simulated maximum likelihood, similarly as in [14]; see also their online appendix for details. Estimation is done using Fortran. The code is a modified version of the code developed by Bhalotra and van Soest.

Table 2 presents goodness of fit measures of some alternative specifications of the model. Panel 1 refers to the benchmark model discussed above (Eqs 1–4). Panel 2 is the same model without any unobserved heterogeneity terms. Panel 3 replaces the birth interval and the fertility equation by one censored regression equation, so that not having another birth is modelled as a birth interval extending beyond the reproductive age limit (or the observation window), see Section 3. Panel 4 refers to the benchmark model with logistic errors instead of normally distributed errors.

**Table 2:** Log likelihoods (LL), Akaike Information Criterion (AIC) and Bayesian Information Criterion (BIC) of alternative model specifications.

	icddr,b area	Comparison area
Benchmark model Section 3 (88 parameters)	LL = -26011.47 AIC = 52198.94 BIC = 26407.88	LL = -29088.21 AIC = 58352.38 BIC = 29485.08
Model without unobserved heterogeneity (81 parameters)	LL = -26149.81 AIC = 52461.62 BIC = 26514.69	LL = -29188.92 AIC = 58539.84 BIC = 29554.24
Model without fertility equation (64 parameters)	LL = -28816.73 AIC = 57761.46 BIC = 29105.03	LL = -32875.038 AIC = 65878.08 BIC = 33163.69
Model with logistic errors in the mortality equations (88 parameters)	LL = -26017.81 AIC = 52211.62 BIC = 26414.22	LL = -29090.59 AIC = 58357.18 BIC = 29487.48

The number of parameters is the total number of estimated parameters for each model. For the benchmark model, this is 88: 80 coefficients on the explanatory variables (including the constant) in each equation (mortality equation for first born: 15; mortality equation for later borns: 21; birth interval equation: 22; fertility equation: 22), 6 parameters determining the joint distribution of the three unobserved heterogeneity terms in Eqs 1, 3 and 4, the parameter *θ* in Eq (2), and the standard deviation of the error term in Eq (3). The other models are less rich and set some parameters to zero. *Source*: Matlab DSS data

The Akaike Information Criterion (AIC; number of parameters − log likelihood) and the Bayes Information Criterion (BIC; −2 log likelihood + (number of parameters) x log (number of observations) both indicate that the benchmark model in panel 1 has the best fit (lowest AIC and BIC value). This is the complete model of the previous section, which simultaneously estimates equations for birth interval, the probability to have another child (fertility equation), and for mortality of the first born and later born children, and includes unobserved heterogeneity terms in each of the equations.

Replacing normally distributed errors by logistic errors (panel 4) gives slightly higher AIC and BIC, and very similar results in terms of parameter estimates and simulation outcomes (which are available upon request). Panel 2 gives substantially larger (that is, inferior) AIC or BIC values, emphasizing the importance of incorporating unobserved heterogeneity and accounting for the simultaneous nature of the various mechanisms, rendering separate estimation of each equation inconsistent due to endogeneity. Finally, the model in which fertility and birth spacing are captured by only one equation (panel 3), seems intuitively more appealing (see section 3), but is clearly outperformed by the benchmark model in terms of goodness of fit. Still, most estimates of the simpler models (see panel 2 and panel 3) are qualitatively similar to those of the benchmark model.

### Estimation results benchmark model

We focus on the results for the comparison area without health and family planning interventions. Estimation results for the complete model (Eqs 1, 2, 3 and 4) in this area are presented in Tables 3 and 4. Estimates for the icddr,b area are presented in the S1 and S2 Tables of supportive information section and we discuss the main differences with the comparison area at the end of this section. Moreover, in the simulations in the next section we will also consider the implications of the differences in the estimates.

**Table 3:** Parameter estimates based on benchmark model in comparison area, n = 32,366.

Variable	Infant mortality later borns (Eq (1))		Infant mortality first borns (Eq (2))		Log birth interval (Eq (3))		Fertility equation (Eq (4))	
	estimate	s.e	estimate	s.e	estimate	s.e	estimate	s.e
**Preceding birth interval (log)**	-1.7239**	0.4191	**-**	-	-	-	-	-
**Preceding birth interval square (log)**	0.2094**	0.0571	**-**	-	-	-	-	-
**Log birth interval x Previous sibling died**	0.0648	0.1157	**-**	-	-	-	-	-
**Previous sibling died**	-0.2703	0.3712	**-**	**-**	-0.6107**	0.0147	-0.2092*	0.0991
**Male child**	0.0111	0.0309	0.0648	0.0352	-0.0306**	0.0092	-0.0197	0.0485
**Muslim**	-0.0503	0.0516	0.0082	0.0597	0.0090	0.0111	0.3869**	0.1001
**Birth order of the child**	-0.1512*	0.0583	**-**	-	0.0746**	0.0160	0.3148**	0.1015
**Birth order square**	0.0199*	0.0069	**-**	-	-0.0136**	0.0018	-0.0173*	0.0069
**Mother’s birth cohort: 1966–1970**	-0.1516**	0.0400	0.0030	0.0585	0.0461**	0.0090	-0.1730*	0.0672
**1971–1975**	-0.3055**	0.0486	-0.0085	0.0625	0.1072**	0.0109	-0.5095**	0.0991
**After 1975**	-0.5461**	0.0619	-0.1789**	0.0647	0.1554**	0.0131	-0.9052**	0.1573
**Mother’s age at birth**	-0.0321	0.0333	-0.1507**	0.0386	0.0207*	0.0082	-0.0613	0.0468
**Mother’s age at birth square**	0.0004	0.0006	0.0028**	0.0008	-0.0002	0.0002	-0.0028**	0.0009
**Mother’s education some primary**	0.0096	0.0400	-0.1373*	0.0466	0.0565**	0.0083	-0.1940*	0.0711
**Mother’s education at least some secondary**	-0.0896	0.0543	-0.2984**	0.0536	0.1247**	0.0101	-0.5045**	0.1017
**Father’s education some primary**	-0.0286	0.0393	-0.0569	0.0432	-0.0171*	0.0081	0.1156	0.0664
**Father’s education at least some secondary**	-0.1312*	0.0500	-0.0529	0.0495	0.0066	0.0089	-0.0957	0.0770
**Father’s occupation is day labourer**	0.1239*	0.0452	0.0659	0.0529	-0.0440**	0.0104	-0.4155**	0.0862
**Source of drinking water: tubewell /piped**	-0.0194	0.0395	-0.0731	0.0431	0.0243**	0.0080	-0.1453*	0.0606
**Distance to health facility (km)**	0.0064	0.0039	0.0158**	0.0043	-0.0009	0.0008	0.0245**	0.0068
**At least one boy surviving**	-	-	**-**	**-**	0.1226**	0.0160	-1.2778**	0.1699
**At least one girl surviving**	-	-	**-**	**-**	0.0723**	0.0161	-1.2930**	0.1641
**Number of boys surviving in excess of 1**	-	-	**-**	**-**	0.0764**	0.0143	-1.1801**	0.1503
**Number of girls surviving in excess of 1**	-	-	**-**	**-**	0.0197	0.0136	-0.6347**	0.1104
**Constant**	2.8594**	0.8554	-0.6178	0.4549	3.0370**	0.0982	6.9565**	0.9225
**Std. deviation error term**	-	-	-	-	0.4356**	0.0027	-	-

Notes

* 2 < t-value < 3

** t-value ≥ 3

Reference category: gender is female, religion is Muslim, mother and father have no education, father is not day-labourer, source of drinking water is tube-well/pipewater, and mother’s birth cohort before 1966. No education = 0 years of schooling, some primary education = 1–5 years of schooling, and at least some secondary education = 6 or more years of schooling

**Table 4:** Benchmark model, comparison area: Estimated covariance structure of mother specific unobserved heterogeneity terms.

	Mortality	Birth interval	Fertility
*Covariance matrix*			
Mortality	0.0625**		
Birth interval	-0.0002	0.007**	
Fertility	-0.188**	-0.088**	2.306**
*Correlation matrix*			
Mortality	1		
Birth interval	-0.012	1	
Fertility	-0.495**	-0.698**	1

** t-value>3

### Infant mortality equations

The parameter estimates of Eq (1) are presented in the left hand panel of Table 3. The estimated quadratic pattern (the first two parameters) implies that, keeping other covariates and unobserved mother specific factors constant, if the previous child survived its infancy, the mortality probability falls with the length of the birth interval as long as the birth interval is less than 63.3 months, i.e., for most of the birth interval range (cf. Fig 3). If the previous child died, mortality falls with birth interval length for intervals up to 52.5 months, still much beyond the median. The difference between the two patterns (the interaction term *log birth interval x previous sibling died*) is not significant. Keeping the birth interval and other covariates constant, the effects of mortality of the previous sibling are insignificant (both the main effect of *Previous sibling died* and its interaction with the *log birth interval*).

The estimated coefficients on the covariates are in line with those in [35]. We focus on the significant ones. Mortality risk is U-shaped in birth order, which is in line with the raw data. We find strong cohort effects, with much lower mortality risk for the younger cohorts of mothers (the reference group is mothers born before 1966). There is some evidence of mortality reducing effects of socio-economic status: Secondary schooling of the father significantly reduces infant mortality risk. Children of fathers of low occupational status (day labourers) have a significantly larger probability of mortality than other children.

Parameter estimates for mortality of the first-born child (Eq (2)) are presented in the second panel of Table 3. Again, infants of the youngest cohorts of mothers face substantially less mortality risk than other children. In this case we find a significant and U-shaped pattern of the mother’s age at birth, with a minimum mortality risk at age 27. As for later born children, higher socio-economic status protects against infant mortality, but this time the mother’s education level is the significant indicator of that. Finally, the mortality risk of the first child is significantly higher for families living farther away from the nearest health clinic, something we did not find for later born children.

### Birth-spacing equation

The third panel of Table 3 reports the estimates of the birth spacing equation. Since the dependent variable is the log of the birth interval, parameters must be interpreted in terms of percentage changes in the expected length of the birth interval. In the comparison area, death at infancy of the previous child shortens the subsequent birth interval by about 46% (exp(-0.6107)-1), consistent with the replacement hypothesis and existing findings [2]. Having at least one boy has a stronger positive effect on the birth interval than having a girl. The same applies to each additional boy. For example, the ceteris paribus difference between the next birth interval of families with one (surviving) boy and families with one girl is 5.2% (exp(0.1226–0.0723)-1). If a family already has one boy and one girl and the third child is a boy (and all children survive), the predicted length of the birth interval after this third birth is 5.8% (exp(0.0764–0.0197)-1) longer than if the third child is a girl.

Birth intervals shorten with birth order as in, for example, [36]. They are longer for the younger birth cohorts of mothers, which may explain part of the reduction in fertility over time. In the comparison area, birth interval length increases with the mother’s age at the previous birth over the whole reproductive age range. This is in line with the negative effect of maternal age on the hazard rate of a new conception found by ([37], Table 2). Birth intervals increase with the mother’s education level, in line with the positive relation between birth intervals and socioeconomic status. This is in line with the finding that the use of contraception is more common among higher socioeconomic status groups [38]. Mothers in villages with more hygienic sources of drinking water tend to have longer birth intervals, probably since the source of drinking water is an index of the level of development in the village, and more developed villages make more use of family planning.

### Equation for a next birth

The right hand panel of Table 3 presents the estimates of Eq (4) determining the probability of having another child after each birth. The most important variables in this equation concern family composition and these effects are qualitatively in line with those of the birth interval equation, as expected. Having at least one son or at least one daughter substantially and significantly reduces the probability to have further children. Additional sons substantially reduce the tendency to have more children, but additional girls have a much smaller effect. The likelihood to have another birth falls with the level of education of both parents, with a larger effect of mother’s education. Muslim families show a higher tendency to have more births than non-Muslims. Keeping other variables (including the number and gender composition of surviving children, the mother’s age, and survival of the previous child), the desire for continued births rises with birth order, implying that earlier infant deaths increase desired fertility. Younger mothers are less likely to have more births than older mothers (keeping family composition and other variables constant). There are strong cohort differences, implying that the younger cohorts less often want more children. Families in villages without hygienic source of drinking water or living far away from a health centre have a larger probability to have another child. A potential explanation is that mothers in villages with fewer facilities anticipate higher chances that some of their children will die (after infancy) and want to guarantee a large enough number of surviving children [20]. In both areas day labourers have smaller chances to have more children, perhaps because their unstable labour market position makes it difficult for them to support a larger family. On the other hand, one might expect them to plan to have more children, accounting for the larger probability that one of them may die (“hoarding”), Apparently, the latter effect (if it exists) is dominated by the first.

### Unobserved heterogeneity

The estimates of the covariance matrix of the three unobserved heterogeneity terms are given in Table 4. The heterogeneity terms in all three equations are statistically significant. In the comparison area, mother specific effects in the mortality equation explain 5.88% of the total unsystematic variation in infant mortality:

$$(Var(\alpha_{mi})/[Var(\alpha_{mi})+Var(u_{mit})])=0.0625/(0.0625+1)=0.0588$$

For the birth spacing equation the idiosyncratic noise terms have an estimated standard deviation 0.4356 (see bottom of Table 3). The unobserved heterogeneity term has estimated variance 0.007 (Table 4), so that unobserved heterogeneity explains only 3.60% of the total unsystematic variance in this equation:

$$(Var(\alpha_{bi})/[Var(\alpha_{bi})+Var(u_{bit})])=0.007/(0.007+0.4356^{2})=0.036$$

The small correlations between unobserved heterogeneity in the mortality and birth interval equations (see correlation matrix in bottom part of Table 4) confirm that hoarding does not play an important role (see Section 1). The heterogeneity terms in the equation for having another child explain almost 70% of the total unsystematic variation (see Table 4) in that equation:

$$(Var(\alpha_{fi})/[Var(\alpha_{fi})+Var(u_{fit})])=2.306/(2.306+1)=0.6975$$

A large negative correlation is observed between unobserved heterogeneity in birth interval and fertility equations, confirming that both equations are strongly related: mothers who desire many children also tend to use shorter birth intervals. This is consistent with the target fertility model of Wolpin [4] and in line with the findings in [14]. The correlation between the individual effects in the mortality equation and the fertility equation is significantly negative in the comparison area. It suggests there is some unobserved factor that increases mortality and at the same time reduces fertility. Although we include several indicators of socio-economic status in the regressions, this could be an unobserved component like income or wealth. Low income may reduce the quality of nutrition or hygienic circumstances and thus increase mortality. At the same time, a lack of resources to support many children may reduce fertility.

### Results for the icddr,b area

The results for the icddr,b area, where access to information and medical treatment is easier than in the comparison area, are presented in the S1–S2 Tables of supportive information. In many respects these results are qualitatively similar to those in the comparison area. The main difference is in the role of the birth interval and lagged mortality on mortality (Eq (2)). Both lagged mortality and the interaction of lagged mortality with the preceding birth interval are significant (with coefficients -1.9904 and 0.5471, respectively). The results imply that for short birth intervals, previous infant mortality has a negative effect on mortality on the next child, possibly due to learning. This effect, however, fades out for longer birth intervals. Moreover, the magnitude of the negative birth interval effect on mortality is larger in the comparison area (where the effect is always substantial and negative, except at extremely large birth intervals) than in the icddr,b area (where the effect turns zero or even positive for large birth intervals).

## 5. Simulations

To demonstrate the importance of the causal mechanisms between birth spacing, having a next birth, and infant mortality, we performed some simulations, in a similar way as ([14], Table 3). The simulations exploit the main feature of our joint model: the fact that it incorporates various mechanisms that lead to associations between birth spacing, the number of births, and mortality outcomes, accounting for the effects of endogeneity in the timing of births (and therefore also age at birth etc.), birth intervals, and mortality risks.

The simulations start from the observed covariates (including, for example, date of first birth) for the actual sample of mothers. For each mother, we generated unobserved heterogeneity terms, error terms, and new outcomes (the dependent variables in our model) using the estimated parameters of each equation. The outcomes were generated recursively, using the timing of the events as sketched in Section 4. For example, for a given mother, we take the date of first birth as given and first generate the mortality outcome of the first child (using Eq (2)). Given simulated mortality, we then generated the outcome whether or not a second birth takes place (Eq (4)). If it does, we then generate a birth interval, and update calendar time and age of the mother at her second birth. Given these variables, other covariates, and the previous mortality outcome, we then generate the mortality outcome of the second born child, etc. In this way we generate complete birth, birth spacing, and mortality patterns for all mothers in the sample. To reduce simulation variance, this is repeated 25 times for each mother.

Table 5 shows the results of several simulations. Column 1 summarizes the outcomes according to the benchmark simulation where all mechanisms incorporated in the model are active. As expected, this column reproduces several features of the raw data, such as the differentials in infant mortality rates and median birth intervals between the two areas.

**Table 5:** Simulations.

Icddr,b area	1	2	3	4
Infant mortality/1000 livebirths	51.8	-0.27	4.85	1.63
Median birth interval (months)	43.12	5.89	-0.20	3.86
Mean number of births (fertility)	2.43	-2.20	0.01	-3.32
Mean number of survivors children	2.31	-2.18	-0.26	-3.40
**Comparison area**				
Infant mortality/1000 livebirths	68.500	-3.57	1.56	0.43
Median birth interval (months)	35.95	6.35	-0.11	3.18
Mean number of births (fertility)	2.75	-3.72	-0.35	-5.68
Mean number of survivors susurvivorssurvchildrenvor children	2.56	-3.47	-0.46	-5.71

Notes: Column 1 presents simulated outcomes for the benchmark model. Columns 2–4 show percentage deviations from the benchmark outcomes that arise when selected mechanisms are “switched off” as follows

Column 2: no effect of infant mortality on birth interval or probability of having another child

Column 3: no direct effect of lagged mortality on mortality

Column 4: birth spacing and family planning as if all children are boys (no gender preference in birth intervals or probability of having another child)

The other columns present percent deviations from the benchmark for scenarios in which some behavioural or non-behavioural mechanisms are “switched off.” Column 2 switches off the replacement effects of infant mortality on both birth intervals and the probability of having another child. The estimates imply that families respond to infant mortality by shortening the next birth interval and increasing the number of births, and this is incorporated in the benchmark simulation in column 1. The simulation in column 2 produces the counterfactual outcomes that would arise if families would space their births and plan the number of births as if every child survived its infancy. The results show that this increases median birth interval length by 5.89% and 6.35% in the two areas, in line with the replacement effects on birth intervals found in other studies.

In the comparison area, the total replacement effect resulting from the infant mortality rate of 68.5 per 1000 live births is an increase in the number of births by 3.72%, that is, 0.54 births for every infant that died (37.2/68.5). In the icddr,b area, the replacement effect is an increase of the total number of births by 2.20%, or 0.42 births for every infant that died. The larger effect in the comparison area is mainly due to the larger response of the number of births to the family composition variables in that area. Because of the longer birth intervals and the reduction in births, eliminating the replacement effects also has an indirect effect on infant mortality in the comparison area: it falls by 3.57% (2.45 per 1000 live births). In the icddr,b area, this indirect effect is much smaller (-0.27%).

Column 3 shows what happens if the direct effect of mortality of the previous child on survival chances is eliminated. (It does not eliminate replacement effects.) Since this direct effect was negative in both areas, eliminating it increases infant mortality: by 4.85% (2.51 infant deaths per 1000 live births) in the icddr,b area and by 1.56% (1.07 per 1000 live births) in the comparison area. The difference between the two areas is due to the larger negative state dependence effect in the icddr,b area for short birth intervals, which can be explained by learning. Eliminating such a learning effect would increase infant mortality among children whose previous sibling died. Because of replacement behaviour, the larger infant mortality rates indirectly also shorten birth intervals and increase the probability to have a next birth, leading to higher fertility, but Table 5 shows that the effects on the numbers of children (both children born and children surviving infancy) are small, particularly in the icddr,b area with the smaller replacement effects.

The final simulation (column 4) illustrates the importance of gender composition in the number of births and birth spacing. We suppress gender preference by simulating counterfactual birth and birth spacing processes, assuming that families behave as if all their children were boys. This would lengthen the median birth interval by 3.86% in the icddr,b area and by 3.18% in the comparison area, and it would reduce total fertility by 3.32% in the icddr,b area and by 5.68% in the comparison area. The interpretation is that for many families, not only the target family size matters for family planning, but also the target number of sons (“son preference”). Although these behavioural changes would reduce the infant mortality rates for higher order births, the ultimate effect on the infant mortality rate is positive. This is due to a composition effect: since the number of higher order births is reduced, the weight of relatively risky first births in the total infant mortality rate has increased.

## 6. Discussion

We analysed infant mortality and fertility behaviour with an important role of short birth intervals, distinguishing causal mechanisms from unobserved heterogeneity and reverse causality by using dynamic panel data techniques, building on the model of Bhalotra and van Soest. We used prospective data covering two rural areas in Matlab, Bangladesh: a treatment area with extensive health and family planning services and a comparison area with the standard health services provided by the government.

The main goal was to explore the causal mechanisms between infant deaths and total fertility, and how birth spacing shapes this relationship. Comparing the findings in the two areas reveals several significant differences resulting from the interventions [39]. We also tried using dummies for whether specific interventions were introduced at the time of birth, but these were not significant so that we are not able to analyse the efficiency of specific health or family planning interventions.

Controlling for birth spacing, unobserved heterogeneity, and a large set of socio-demographic covariates, we found insignificant negative state dependence in the comparison area but significant negative state dependence in the treatment area in case of short birth intervals. In other words, a child born after a short birth interval has higher chances to survive its infancy if the previous sibling died than if it survived. This can be due to learning, stimulated by the extensive family health services in the treatment area. This finding is unique among studies of infant mortality. For example, [23] found higher risks of death during infancy in Matlab if the previous sibling also died during infancy. [15] found positive state dependence in the neonatal as well as the post-neonatal period. For India, [26] found that infant death of the previous sibling increases the likelihood of infant death by between 2.2 and 9.2 percent points. Similarly, [31] found a positive scarring effect of 4.8 percent points for Kenya. These studies do not control for birth intervals. In an earlier study ([35], Table 5), we also found negative state dependence when keeping preceding birth intervals constant, but the negative effect is about two to three times larger in the current study, which emphasizes the importance of allowing for the endogeneity of birth-spacing in the model (namely correlation between the unobservables driving mortality and fertility). This is in line with [40] where it was already discussed how strong positive correlation between mortality and fertility biases the regression estimates and possible corrections.

We find evidence of causal effects in two directions: a short preceding birth interval reduces survival chances of infants, and an infant death increases the probability of a next birth and shortens the time until the next birth (replacement behaviour). For the comparison area we estimate that, as a result of replacement, 0.54 children are born for each infant death, compared to 0.42 children in the icddr,b area with additional health and family planning services. This finding is in line with [13], where a replacement effect of 0.55 births for each infant death was found.

The negative effect of infant mortality on the birth interval is large in comparison area. The literature provides evidence of biological effects through truncating the lactation period providing protection against fertility after a child death [41]. It is worth to note here that our replacement measure includes both volitional and biological replacement effects [13] and we cannot disentangle different replacement strategies discussed several replacement strategies.

We find that in the comparison area, mortality risk falls with birth interval length over almost the complete range. In the icddr,b area, however, higher mortality risk is found after a long birth interval, particularly after an infant death, suggesting that after an infant death and a long interval the mother may behave as a mother who gives birth to her first child [42].

Estimates of reproductive behaviour are consistent with gender preference: having more surviving boys significantly reduces the probability of having a next child and this effect is strongest in the comparison area, in line with, e.g., [43]. In both areas, day labourers have smaller chances to have more children, which is not in line of replacement hypothesis. This is perhaps because their unstable labour market position makes it difficult for them to support a larger family.

Concerning policies targeted at achieving the sustainable development goals to improve reproductive health and reduce child mortality, the difference between the findings for the two areas highlight the important role of extensive maternal and child health interventions. Comprehensive health infrastructure, providing extensive health services and health and family planning information in the treatment area, strengthens learning effects that can reduce mortality risk. Moreover, it changes family planning and reduces the size of replacement effects after an infant death as well as the additional number of children due to boy preference.

Our study has several limitations that were already briefly discussed in the modelling and data sections. We have used a selected sample, excluding families who migrated into or out of the area during the relevant time period. Lack of available data prevented us from performing a sensitivity analysis to investigate the effect of the selection procedure. On the other hand, results in ([13], p. 395) suggest that the replacement effects do not vary substantially with the sample selection. Stillbirths are discarded, and disentangling stillbirths from other mechanisms that may cause a large birth interval seems a worthwhile extension of the model. Moreover, it would be interesting to incorporate the use of contraception as an explicit tool for family planning ([44], for a possible way of modelling this), but the current data do not allow for this. Local community effects were not considered either, due to the additional complexity this would involve. Finally, we only consider infant mortality, which is hardly affected by the next birth interval (which is typically longer than a year). An extension considering child mortality (during the first five years of life) in which not only the preceding but also the subsequent birth interval would play a role, seems another challenging extension of the current modelling framework.

## Supporting information

S1 Table Parameter estimates based on benchmark model in icddr,b area, n = 31,968. (DOC)

S2 Table Benchmark model, icddr,b area: Estimated covariance structure of mother specific unobserved heterogeneity terms. (DOC)

S3 Table Parameter estimates based on logistic regression model in comparison area, n = 32,366. (DOC)

S4 Table Parameter estimates based on logistic regression model in icddr,b area, n = 31,968. (DOC)

S5 Table Logistic model, comparison area: Estimated covariance structure of mother specific unobserved heterogeneity terms. (DOC)

S6 Table Logistic model, icddr,b area: Estimated covariance structure of mother specific unobserved heterogeneity terms. (DOC)

S7 Table Simulations based on logistic models. (DOC)
