# Supplementary material for: Relationships between infant mortality, birth spacing and fertility in Matlab, Bangladesh
Source: PLoS One. 2018 Apr 27;13(4):e0195940. doi: 10.1371/journal.pone.0195940 (PMC5922575; doi:10.1371/journal.pone.0195940)
Supplement: S1 Table — (DOC) [file pone.0195940.s001.doc]

**S1 Table S1: Parameter estimates based on benchmark model in icddr,b area, n=31,968**

| **Variable** | **Infant mortality later borns (Eq. (1))** | | **Infant mortality first borns (Eq. (2))** | | | **Log birth interval**  **(Eq. (3))** | | | **Fertility equation (Eq. (4))** | | |  |
| --- | --- | --- | --- | --- | --- | --- | --- | --- | --- | --- | --- | --- |
|  | estimate | s.e | | estimate | s.e | | estimate | s.e | | estimate | s.e | |
| **Preceding birth interval (log)** | -2.7871** | 0.4772 | | **-** | - | | - | - | | - | - | |
| **Preceding birth interval square (log)** | 0.3565** | 0.0644 | | **-** | - | | - | - | | - | - | |
| **Log birth interval * Previous sibling died** | 0.5471** | 0.1384 | | **-** | - | | - | - | | - | - | |
| **Previous sibling died** | -1.9904** | 0.4637 | | **-** | **-** | | -0.6741** | 0.0178 | | -0.15572 | 0.1004 | |
| **Male child** | 0.0352 | 0.0399 | | 0.1249** | 0.0372 | | -0.0104 | 0.0103 | | -0.0462 | 0.0462 | |
| **Muslim** | -0.0275 | 0.0604 | | -0.0022 | 0.0506 | | -0.0145 | 0.0105 | | 0.6076** | 0.0787 | |
| **Birth order of the child** | 0.0494 | 0.1091 | | **-** | - | | 0.1136** | 0.0219 | | -0.3857* | 0.1640 | |
| **Birth order square** | -0.01327 | 0.0152 | | **-** | - | | -0.0228** | 0.0026 | | 0.0100 | 0.0089 | |
| **Mother’s birth cohort: 1966-1970** | -0.0213 | 0.0548 | | -0.1351* | 0.0572 | | 0.0659** | 0.0098 | | 0.0418 | 0.0473 | |
| **1971-1975** | -0.1513* | 0.0674 | | -0.1897** | 0.0612 | | 0.1556** | 0.0116 | | 0.1051 | 0.0720 | |
| **After 1975** | -0.1878* | 0.0807 | | -0.4205** | 0.0635 | | 0.2320** | 0.0130 | | 1.2814 | 0.6862 | |
| **Mother’s age at birth** | -0.1260** | 0.0371 | | -0.0845* | 0.0358 | | 0.0262** | 0.0065 | | 0.0008 | 0.0333 | |
| **Mother’s age at birth square** | 0.0020** | 0.0006 | | 0.0013 | 0.0008 | | -0.0004* | 0.0001 | | -0.0026 | 0.0007 | |
| **Mother’s education some primary** | -0.0616 | 0.0537 | | -0.1832** | 0.0491 | | 0.0372** | 0.0091 | | 0.0331 | 0.0539 | |
| **Mother’s education at least some secondary** | -0.0697 | 0.0697 | | -0.2955** | 0.0575 | | 0.0565** | 0.0107 | | -0.3843** | 0.0790 | |
| **Father’s education some primary** | 0.0604 | 0.0506 | | 0.0241 | 0.0457 | | -0.0054 | 0.0088 | | 0.0229 | 0.0522 | |
| **Father’s education at least some secondary** | -0.2305** | 0.0684 | | -0.0914 | 0.0543 | | 0.0035 | 0.0098 | | -0.1249* | 0.0603 | |
| **Father’s occupation is day labourer** | 0.1271* | 0.0636 | | 0.0225 | 0.0575 | | -0.0046 | 0.0121 | | -0.5451** | 0.0790 | |
| **Source of drinking water: tubewell /piped** | -0.1767* | 0.0633 | | -0.0289 | 0.0520 | | 0.0414** | 0.0101 | | -0.1205* | 0.0554 | |
| **Distance to health facility (km)** | -0.0002 | 0.0227 | | 0.0150 | 0.0176 | | 0.0042 | 0.0037 | | -0.0216 | 0.0181 | |
| **At least one boy surviving** | - | - | | **-** | **-** | | 0.1726** | 0.0203 | | -0.5969** | 0.1568 | |
| **At least one girl surviving** | - | - | | **-** | **-** | | 0.1099** | 0.0198 | | -0.5211** | 0.1537 | |
| **Number of boys surviving in excess of 1** | - | - | | **-** | **-** | | 0.0978** | 0.0191 | | -0.3367* | 0.1443 | |
| **Number of girls surviving in excess of 1** | - | - | | **-** | **-** | | 0.0325 | 0.0186 | | 0.0307 | 0.1403 | |
| **Constant** | 5.4656** | 0.9774 | | -0.1035 | 0.4232 | | 3.0807** | 0.0801 | | 4.4781** | 0.5415 | |
| **Std. deviation error term** | - | - | | - | - | | 0.4422** | 0.0029 | | - | - | |

Notes:* 2 < t-value < 3; ** t-value ≥ 3

Reference category: gender is female, religion is Muslim, mother and father have no education, father is not day-labourer, source of drinking water is tube-well/pipewater, and mother’s birth cohort before 1966. No education=0 year of schooling, some primary education=1-5 years of schooling, and at least some secondary education=6 or more years of schooling
