# Supplementary material for: Relationships between infant mortality, birth spacing and fertility in Matlab, Bangladesh
Source: PLoS One. 2018 Apr 27;13(4):e0195940. doi: 10.1371/journal.pone.0195940 (PMC5922575; doi:10.1371/journal.pone.0195940)
Supplement: S2 Table — (DOC) [file pone.0195940.s002.doc]

**S2 Table S2: Benchmark model, icddr,b area: Estimated covariance structure of mother specific unobserved heterogeneity terms**

|  | **Mortality** | **Birth interval** | **Fertility** |
| --- | --- | --- | --- |
| *Covariance matrix* |  |  |  |
| Mortality | 0.301** |  |  |
| Birth interval | -0.012 | 0.017** |  |
| Fertility | 0.189 | -0.099** | 0.793** |
| *Correlation matrix* |  |  |  |
| Mortality | 1 |  |  |
| Birth interval | -0.167 | 1 |  |
| Fertility | 0.386 | -0.856** | 1 |

** t-value>3
