# Supplementary material for: Relationships between infant mortality, birth spacing and fertility in Matlab, Bangladesh
Source: PLoS One. 2018 Apr 27;13(4):e0195940. doi: 10.1371/journal.pone.0195940 (PMC5922575; doi:10.1371/journal.pone.0195940)
Supplement: S3 Table — (DOC) [file pone.0195940.s003.doc]

**S3 Table S3: Parameter estimates based on logistic regression model in comparison area, n=32,366**

| **Variable** | **Infant mortality later borns (Eq. (1))** | | **Infant mortality first borns (Eq. (2))** | | | **Log birth interval**  **(Eq. (3))** | | | **Fertility equation (Eq. (4))** | | |  |
| --- | --- | --- | --- | --- | --- | --- | --- | --- | --- | --- | --- | --- |
|  | estimate | s.e | | estimate | s.e | | estimate | s.e | | estimate | s.e | |
| **Preceding birth interval (log)** | -3.5420** | 0.8482 | | **-** | - | | - | - | | - | - | |
| **Preceding birth interval square (log)** | 0.4271** | 0.1162 | | **-** | - | | - | - | | - | - | |
| **Log birth interval * Previous sibling died** | 0.1642 | 0.2315 | | **-** | - | | - | - | | - | - | |
| **Previous sibling died** | -0.6865 | 0.7337 | | **-** | **-** | | -0.6104** | 0.0147 | | -0.1490 | 0.0831 | |
| **Male child** | 0.0256 | 0.0648 | | 0.1291 | 0.0689 | | -0.0305** | 0.0092 | | -0.0281 | 0.0431 | |
| **Muslim** | -0.1110 | 0.1058 | | 0.0080 | 0.1146 | | 0.0095 | 0.0111 | | 0.3347** | 0.0793 | |
| **Birth order of the child** | -0.2793* | 0.1230 | | **-** | - | | 0.0744** | 0.0162 | | 0.2988** | 0.0788 | |
| **Birth order square** | 0.0357* | 0.0148 | | **-** | - | | -0.0136** | 0.0017 | | -0.0122* | 0.0060 | |
| **Mother’s birth cohort: 1966-1970** | -0.3210** | 0.0818 | | 0.0035 | 0.1115 | | 0.0461** | 0.0091 | | -0.1480* | 0.0552 | |
| **1971-1975** | -0.6349** | 0.1015 | | -0.0164 | 0.1197 | | 0.1075** | 0.0109 | | -0.4462** | 0.0768 | |
| **After 1975** | -1.1541** | 0.1333 | | -0.3630* | 0.1257 | | 0.1548** | 0.0131 | | -0.7850** | 0.1171 | |
| **Mother’s age at birth** | -0.0756 | 0.0689 | | -0.2871** | 0.0728 | | 0.0215* | 0.0081 | | -0.0356 | 0.0390 | |
| **Mother’s age at birth square** | 0.0011 | 0.0012 | | 0.0052** | 0.0015 | | -0.0003 | 0.0002 | | -0.0027** | 0.0007 | |
| **Mother’s education some primary** | 0.0163 | 0.0839 | | -0.2649* | 0.0904 | | 0.0562** | 0.0083 | | -0.1419* | 0.0565 | |
| **Mother’s education at least some secondary** | -0.2059 | 0.1173 | | -0.5952** | 0.1070 | | 0.1245** | 0.0101 | | -0.3950** | 0.0769 | |
| **Father’s education some primary** | -0.0730 | 0.0831 | | -0.1127 | 0.0846 | | -0.0165* | 0.0081 | | 0.0893 | 0.0555 | |
| **Father’s education at least some secondary** | -0.2665* | 0.1066 | | -0.1051 | 0.0990 | | 0.0073 | 0.0090 | | -0.1055 | 0.0645 | |
| **Father’s occupation is day labourer** | 0.2593* | 0.0927 | | 0.1234 | 0.1017 | | -0.0442** | 0.0104 | | -0.3416** | 0.0664 | |
| **Source of drinking water: tubewell /piped** | -0.0314 | 0.0810 | | -0.1305 | 0.0828 | | 0.0242** | 0.0081 | | -0.1315* | 0.0517 | |
| **Distance to health facility (km)** | 0.0143 | 0.0082 | | 0.0307 | 0.0085 | | -0.0009 | 0.0008 | | 0.0198** | 0.0055 | |
| **At least one boy surviving** | - | - | | **-** | **-** | | 0.1222** | 0.0160 | | -1.1158** | 0.1220 | |
| **At least one girl surviving** | - | - | | **-** | **-** | | 0.0722** | 0.0160 | | -1.1266** | 0.1154 | |
| **Number of boys surviving in excess of 1** | - | - | | **-** | **-** | | 0.0758** | 0.0139 | | -1.0153** | 0.1036 | |
| **Number of girls surviving in excess of 1** | - | - | | **-** | **-** | | 0.0197 | 0.0136 | | -0.5567** | 0.0832 | |
| **Constant** | 6.3721** | 1.7333 | | 1.3942 | 0.8637 | | 3.0285** | 0.0980 | | 5.7367** | 0.6066 | |
| **Std. deviation error term** | - | - | | - | - | | 0.4352** | 0.0027 | | - | - | |

Notes:* 2 < t-value < 3; ** t-value ≥ 3

Reference category: gender is female, religion is Muslim, mother and father have no education, father is not day-labourer, source of drinking water is tube-well/pipewater, and mother’s birth cohort before 1966. No education=0 year of schooling, some primary education=1-5 years of schooling, and at least some secondary education=6 or more years of schooling
