# Supplementary material for: Relationships between infant mortality, birth spacing and fertility in Matlab, Bangladesh
Source: PLoS One. 2018 Apr 27;13(4):e0195940. doi: 10.1371/journal.pone.0195940 (PMC5922575; doi:10.1371/journal.pone.0195940)
Supplement: S4 Table — (DOC) [file pone.0195940.s004.doc]

**S4 Table S4: Parameter estimates based on logistic regression model in icddr,b area, n=31,968**

| **Variable** | **Infant mortality later borns (Eq. (1))** | | **Infant mortality first borns (Eq. (2))** | | | **Log birth interval**  **(Eq. (3))** | | | **Fertility equation (Eq. (4))** | | |  |
| --- | --- | --- | --- | --- | --- | --- | --- | --- | --- | --- | --- | --- |
|  | estimate | s.e | | estimate | s.e | | estimate | s.e | | estimate | s.e | |
| **Preceding birth interval (log)** | -5.3402** | 0.9273 | | **-** | - | | - | - | | - | - | |
| **Preceding birth interval square (log)** | 0.6781** | 0.1258 | | **-** | - | | - | - | | - | - | |
| **Log birth interval * Previous sibling died** | 1.0273** | 0.2631 | | **-** | - | | - | - | | - | - | |
| **Previous sibling died** | -3.6750** | 0.8734 | | **-** | **-** | | -0.6767** | 0.0179 | | -0.1105 | 0.0931 | |
| **Male child** | 0.0752 | 0.0828 | | 0.2507** | 0.0751 | | -0.0098 | 0.0104 | | -0.0475 | 0.0431 | |
| **Muslim** | -0.0454 | 0.1233 | | -0.0126 | 0.1010 | | -0.0142 | 0.0104 | | 0.5502** | 0.0570 | |
| **Birth order of the child** | 0.1004 | 0.2292 | | **-** | - | | 0.1137** | 0.0219 | | -0.2045* | 0.1249 | |
| **Birth order square** | -0.0239 | 0.0320 | | **-** | - | | -0.0224** | 0.0026 | | 0.0060 | 0.0085 | |
| **Mother’s birth cohort: 1966-1970** | -0.0469 | 0.1101 | | -0.2601* | 0.1106 | | 0.0658** | 0.0098 | | 0.0355 | 0.0417 | |
| **1971-1975** | -0.3219* | 0.1381 | | -0.3663** | 0.1196 | | 0.1554** | 0.0115 | | 0.0916 | 0.0639 | |
| **After 1975** | -0.3738* | 0.1618 | | -0.8450** | 0.1259 | | 0.2317** | 0.0131 | | 1.0463* | 0.5136 | |
| **Mother’s age at birth** | -0.2625** | 0.0751 | | -0.1632* | 0.0719 | | 0.0258** | 0.0065 | | -0.0018 | 0.0303 | |
| **Mother’s age at birth square** | 0.0043** | 0.0013 | | 0.0025 | 0.0015 | | -0.0004* | 0.0001 | | -0.0023 | 0.0006 | |
| **Mother’s education some primary** | -0.1244 | 0.1093 | | -0.3603** | 0.0987 | | 0.0376** | 0.0090 | | 0.0295 | 0.0473 | |
| **Mother’s education at least some secondary** | -0.1233 | 0.1422 | | -0.5996** | 0.1179 | | 0.0567** | 0.0107 | | -0.3351** | 0.0651 | |
| **Father’s education some primary** | 0.1179 | 0.1025 | | 0.0487 | 0.0915 | | -0.0056 | 0.0088 | | 0.0201 | 0.0464 | |
| **Father’s education at least some secondary** | -0.4820** | 0.1416 | | -0.1980 | 0.1128 | | 0.0033 | 0.0098 | | -0.1027 | 0.0520 | |
| **Father’s occupation is day labourer** | 0.2735* | 0.1277 | | 0.0401 | 0.1145 | | -0.0045 | 0.0120 | | -0.4749 ** | 0.0575 | |
| **Source of drinking water: tubewell /piped** | -0.3818** | 0.1251 | | -0.0686 | 0.1010 | | 0.0417** | 0.0101 | | -0.1186* | 0.0496 | |
| **Distance to health facility (km)** | 0.0038 | 0.0464 | | 0.0287 | 0.0354 | | 0.0042 | 0.0037 | | -0.0170 | 0.0160 | |
| **At least one boy surviving** | - | - | | **-** | **-** | | 0.1690** | 0.0211 | | -0.6719** | 0.1417 | |
| **At least one girl surviving** | - | - | | **-** | **-** | | 0.1071** | 0.0205 | | -0.6021 ** | 0.1349 | |
| **Number of boys surviving in excess of 1** | - | - | | **-** | **-** | | 0.0944** | 0.0196 | | -0.4188** | 0.1266 | |
| **Number of girls surviving in excess of 1** | - | - | | **-** | **-** | | 0.0293 | 0.0194 | | -0.0828 | 0.1223 | |
| **Constant** | 10.9455** | 1.8858 | | 0.0398 | 0.8518 | | 3.0862** | 0.0799 | | 4.0466** | 0.4227 | |
| **Std. deviation error term** | - | - | | - | - | | 0.4427** | 0.0029 | | - | - | |

Notes:* 2 < t-value < 3; ** t-value ≥ 3

Reference category: gender is female, religion is Muslim, mother and father have no education, father is not day-labourer, source of drinking water is tube-well/pipewater, and mother’s birth cohort before 1966. No education=0 year of schooling, some primary education=1-5 years of schooling, and at least some secondary education=6 or more years of schooling
