# Supplementary material for: Relationships between infant mortality, birth spacing and fertility in Matlab, Bangladesh
Source: PLoS One. 2018 Apr 27;13(4):e0195940. doi: 10.1371/journal.pone.0195940 (PMC5922575; doi:10.1371/journal.pone.0195940)
Supplement: S5 Table — (DOC) [file pone.0195940.s005.doc]

**S5 Table S5: Logistic model, comparison area: Estimated covariance structure of mother specific unobserved heterogeneity terms**

|  | **Mortality** | **Birth interval** | **Fertility** |
| --- | --- | --- | --- |
| **Covariance matrix** |  |  |  |
| Mortality | 0.2807** |  |  |
| Birth interval | -0.0005 | 0.0071** |  |
| Fertility | -0.3618** | -0.0685** | 1.4179** |
| **Correlation matrix** |  |  |  |
| Mortality | 1 |  |  |
| Birth interval | -0.0112 | 1 |  |
| Fertility | -0.5735** | -0.6812** | 1 |

** t-value>3
