# Supplementary material for: Relationships between infant mortality, birth spacing and fertility in Matlab, Bangladesh
Source: PLoS One. 2018 Apr 27;13(4):e0195940. doi: 10.1371/journal.pone.0195940 (PMC5922575; doi:10.1371/journal.pone.0195940)
Supplement: S6 Table — (DOC) [file pone.0195940.s006.doc]

**S6 Table S6: Logistic model, icddr,b area: Estimated covariance structure of mother specific unobserved heterogeneity terms**

|  | **Mortality** | **Birth interval** | **Fertility** |
| --- | --- | --- | --- |
| **Covariance matrix** |  |  |  |
| Mortality | 0.9860** |  |  |
| Birth interval | -0.0199 | 0.0164** |  |
| Fertility | 0.1092 | -0.0886** | 0.4828 |
| **Correlation matrix** |  |  |  |
| Mortality | 1 |  |  |
| Birth interval | -0.1568 | 1 |  |
| Fertility | 0.1572 | -0.9952** | 1 |

** t-value>3
