# Supplementary material for: Relationships between infant mortality, birth spacing and fertility in Matlab, Bangladesh
Source: PLoS One. 2018 Apr 27;13(4):e0195940. doi: 10.1371/journal.pone.0195940 (PMC5922575; doi:10.1371/journal.pone.0195940)
Supplement: S7 Table — (DOC) [file pone.0195940.s007.doc]

**S7 Table S7: Simulations based on logistic models**

| **icddr,b area** | **1** | **2** | **3** | **4** |
| --- | --- | --- | --- | --- |
| Infant mortality/1000 livebirths | 51.8 | -0.45 | 4.67 | 0.44 |
| Median birth interval (months) | 43.4 | 4.48 | 0.94 | 2.58 |
| Mean number of births (fertility) | 2.44 | -2.32 | -0.07 | -3.53 |
| Mean number of survivors children | 2.31 | -2.29 | -0.32 | -3.55 |
| **Comparison area** |  |  |  |  |
| Infant mortality/1000 livebirths | 68.9 | -3.73 | 1.91 | 0.39 |
| Median birth interval (months) | 35.9 | 5.58 | 0.97 | 2.32 |
| Mean number of births (fertility) | 2.76 | -3.99 | -0.39 | -5.62 |
| Mean number of survivors susurvivorssurvchildrenvor children | 2.57 | -3.73 | -0.53 | -5.65 |

Notes: Column 1 presents simulated outcomes for the logit model. Columns 2-4 show percentage deviations from the logistic regression outcomes that arise when selected mechanisms are “switched off” as follows:

Column 2: no effect of infant mortality on birth interval or probability of having another child

Column 3: no direct effect of lagged mortality on mortality

Column 4: birth spacing and family planning as if all children are boys (no gender preference in birth intervals or probability of having another child)
